# An open trial of individualized face-to-face cognitive behavior therapy for psychological distress in parents of children after end of treatment for childhood cancer including a cognitive behavioral conceptualization

**DOI:** 10.7717/peerj.4570

**Published:** 2018-04-11

**Authors:** Lisa Ljungman, Martin Cernvall, Ata Ghaderi, Gustaf Ljungman, Louise von Essen, Brjánn Ljótsson

**Affiliations:** 1 Clinical Psychology in Healthcare, Department of Women’s and Children’s Health, Uppsala University, Uppsala, Sweden; 2 Division of Psychology, Department of Clinical Neuroscience, Karolinska Institutet, Stockholm, Sweden; 3 Pediatric Oncology, Department of Women’s and Children’s Health, Uppsala University, Uppsala, Sweden

**Keywords:** Cognitive behavior therapy, Cancer, Children, Parents, Trial, Posttraumatic stress, Depression

## Abstract

**Objective:**

A subgroup of parents of children who have been treated for childhood cancer report high levels of psychological distress. To date there is no empirically supported psychological treatment targeting cancer-related psychological distress in this population. The aim of the current study was to test the feasibility and preliminarily evaluate the effect of individualized face-to-face cognitive behavior therapy (CBT) for parents of children after the end of treatment for childhood cancer. A secondary aim was to present a cognitive behavioral conceptualization of cancer-related distress for these parents.

**Methods:**

An open trial was conducted where 15 parents of children who had completed successful treatment for cancer three months to five years earlier and who reported psychological distress related to a child’s previous cancer disease were provided CBT at a maximum of 15 sessions. Participants were assessed at baseline, post-intervention, and three-month follow-up using self-reported psychological distress (including posttraumatic stress symptoms (PTSS), depression, and anxiety) and the diagnostic Mini-International Neuropsychiatric Interview. Feasibility outcomes relating to recruitment, data collection, and delivery of the treatment were also examined. Individual case formulations for each participant guided the intervention and these were aggregated and presented in a conceptualization detailing core symptoms and their suggested maintenance mechanisms.

**Results:**

A total of 93% of the participants completed the treatment and all of them completed the follow-up assessment. From baseline to post-assessment, parents reported significant improvements in PTSS, depression, and anxiety with medium to large effect sizes (Cohen’s *d* = 0.65–0.92). Results were maintained or improved at a three-month follow-up. At baseline, seven (47%) participants fulfilled the diagnostic criteria for major depressive disorder and four (29%) fulfilled the criteria for posttraumatic stress disorder, compared to none at a post-assessment and a follow-up assessment. The resulting cognitive behavioral conceptualization suggests traumatic stress and depression as the core features of distress, and avoidance and inactivity is suggested as the core maintenance mechanisms.

**Conclusion:**

The treatment was feasible and acceptable to the participants. Significant improvements in distress were observed during the study. Overall, results suggest that the psychological treatment for parents of children after end of treatment for childhood cancer used in the current study is promising and should be tested and evaluated in future studies.

## Introduction

Survival rates for childhood cancer have increased dramatically and are approaching 80% (Gustafsson, Kogner & Heyman, 2013). Nevertheless, the experience of cancer is associated with numerous stressors for the child and its family (Bruce, 2006; Wakefield et al., 2011) and parents of children diagnosed with cancer report psychological distress such as posttraumatic stress symptoms (PTSS), anxiety, and depression (Dunn et al., 2011; Kazak et al., 2005a; Pöder, Ljungman & von Essen, 2008). For most parents, the distress is higher shortly after the child’s diagnosis and decreases over time (Dolgin et al., 2007; Ljungman et al., 2015; Pöder, Ljungman & von Essen, 2008) to levels comparable to controls two years after end of successful treatment of the child (Pai et al., 2007; Phipps et al., 2005). However, a subgroup (10–44%) reports high levels of distress up to 10 years after end of the child’s treatment (Bruce, 2006; Kearney, Salley & Muriel, 2015; Ljungman et al., 2014). Even though most parents recover from the distress that they experience during the time of the child’s illness and treatment, not all parents do. Thus, it is important to increase knowledge about the mechanisms involved in the development and maintenance of this distress and to develop targeted interventions.

To date, there is no empirically supported treatment (EST) for cancer-related distress in parents of children after the end of treatment for childhood cancer. For parents of children newly diagnosed with cancer, several psychological interventions have been developed and evaluated. In a systematic review and meta-analysis of psychological interventions for parents of chronically ill children (including parents of children with cancer), Law et al. (2014) concluded that across all interventions (cognitive behavior therapy (CBT), problem solving therapy (PST), and systemic therapies), there were no treatment effects regarding parental mental health outcomes. However, PST was shown to improve mental health outcomes for parents of children with newly diagnosed cancer. Similar conclusions were drawn in a systematic review by Eccleston et al. (2015), where psychological therapies (CBT, PST, and family treatment) for parents of children with cancer showed no effects on parental mental health at post-treatment; however, small beneficial effects were reported at follow-up. In addition to the studies included in these systematic reviews and meta-analyses, Cernvall et al. (2015, 2017) developed and evaluated internet-delivered CBT for parents of children with newly diagnosed cancer, and reported positive intervention effects in terms of reductions in PTSS and depression.

For parents of children after the end of treatment for childhood cancer, to the best of our knowledge, only one psychological intervention has been evaluated; the brief intervention called The Surviving Cancer Competently Intervention Program by Kazak et al. (2005b). With the exception of fathers reporting a reduction of intrusive thoughts, evaluations of the treatment showed no significant effects. Taken together, the evidence is emerging for psychological interventions (PST, CBT) for parents of children newly diagnosed with cancer (Cernvall et al., 2015, 2017; Sahler et al., 2005) however, for cancer-related psychological distress in parents after end of treatment, there is to date no EST. Accordingly, researchers in the field have highlighted the need to develop and evaluate psychological interventions for the specific psychological challenges that parents face after end of a child’s cancer treatment, and that such treatments should isolate and target the subgroup of parents that experience high levels of distress (Price et al., 2016; Wakefield et al., 2011). It could be argued that these parents may be at risk for distress in general, and not only distress directly related to the experience of being a parent of child that has been diagnosed with and treated for cancer. However, in order to be able to inform interventions targeting the unique experiences of having a child previously treated for cancer, it is important to investigate the specificity of the cancer-related experiences among these parents. Thus, in the current study we aimed to conceptualize the distress experienced by parents of children previously treated for cancer, which they perceived as related to their child’s disease and treatment.

In order to develop increased understanding of the distress experienced by parents after the completion of their child’s treatment for cancer, the overarching aim of this study was to test the feasibility and preliminarily evaluate a psychological treatment based on cognitive behavioral therapy for these parents. In accordance with best practice, we conducted the study using the Medical Research Council (MRC) guidelines for developing and evaluating complex interventions (Craig et al., 2013). The MRC guidelines emphasize the importance of a gradual approach when developing a new intervention. Furthermore, the guidelines underline that the formal evaluation in a controlled trial should await a theoretical, in-depth understanding of the symptoms addressed by the intervention, and of the likely processes of change. Such an understanding of cancer-related psychological distress in parents of children after the end of treatment for childhood cancer is, however, lacking, as is highlighted by several researchers in the field (Ljungman et al., 2014; Vrijmoet-Wiersma et al., 2008; Wakefield et al., 2011). Kazak et al. (2006; Price et al., 2016) have suggested the pediatric medical traumatic stress model as a conceptual framework for the psychological reactions in children and families across many different types of pediatric injury and illness. However, this model does not include specifications of the mechanisms involved in the development and maintenance of the distress, and the treatment based on the model has not shown an effect in terms of reduction of distress (Kazak et al., 2004). A second aim of the current study was therefore to develop and present a cognitive behavioral conceptualization of cancer-related psychological distress in parents of children after the end of treatment for childhood cancer, upon which a psychological treatment can be based.

## Materials and Methods

The study was an open trial with a within-group design where individual face-to-face CBT was provided to participants at a maximum of 15 sessions. The authors assert that all procedures contributing to this work comply with the ethical standards of the relevant national and institutional committees on human experimentation and with the Helsinki Declaration of 1975, and its most recent revision. The procedures were approved by the regional ethical vetting board of Uppsala (Dnr: 2012/440) and all participants provided written informed consent. The study is reported according to the TREND-statement guidelines for non-randomized clinical trials (Des Jarlais, Lyles & Crepaz, 2004). During the planning of this study, registration of uncontrolled pilot- and feasibility trials was less common in the field of psychology than it is currently. Therefore, this trial was not registered in a WHO-accredited trial registry before initiation. However, the trial was registered after trial completion (trial ID ISRCTN74785895).

### Participants and procedure

Parents were eligible if they had a child who, by that time of consideration, had completed successful cancer treatment at the pediatric oncology center at the Children’s University Hospital in Uppsala three months to five years earlier; spoke Swedish; were able to commute to the departments (located in Uppsala and Västerås) where the CBT treatments were conducted; and confirmed that they experienced psychological distress of any kind that they related to their child’s cancer disease. Parents were excluded if they suffered from a psychiatric disorder in immediate need of treatment (for example severe depression or suicidal ideation) or if they were undergoing psychotherapy. Assessment of severe psychiatric comorbidity was based on a clinical judgment according to participants’ ratings on the Montgomery–Åsberg Depression Rating Scale (MADRS-S; Svanborg & Åsberg, 1994, 2001), and the diagnostic interview called the Mini-International Neuropsychiatric Interview (M.I.N.I.; Sheehan et al., 1998). If both parents of the same child fulfilled the inclusion criteria they were both eligible, but received treatment from different psychologists. For a power of 0.80 to detect a statistically significant difference, assuming a large effect size (*d* = 0.80) and allowing for 25% drop-out, 20 participants were estimated to be included. However, there were no drop-outs at follow-up assessment for the first 15 participants and therefore no more participants were included. The total sample consisted of 15 parents; see Table 1 for participants’ and their children’s characteristics at baseline.

**Table 1:** Baseline characteristics of the participants.

Parents’ characteristics (*n* = 15)	*n* (%)	Mean (SD)
Mothers	8 (53)	
Partner included in study (four couples participated)	8 (53)	
Age		43.5 (5.6, range 35–52)
Marital status		
Married or cohabitant	14 (93)	
Single	1 (7)	
Living with the child’s biological parent	12 (80)	
Completed university studies	6 (40)	
Current occupation status		
Employed	11 (73)	
Unemployed	1 (7)	
Sick-leave	3 (20)	
Previous treatment for psychological ill-health		
Yes^a^	7 (47)	
No	8 (53)	
Children’s characteristics (n = 11)		
Girl	7 (47)	
Age		12.8 (5.7, range 3–21)
Age at diagnosis^b^		9.3 (4.9, range 1–15)
Diagnosis^c^		
Leukemia	4 (36)	
CNS tumor	2 (18)	
Lymphoma	1 (9)	
Sarcoma	1 (9)	
Other malignant disease	3 (27)	
Time since end of treatment (years)		2.2 (1.3, range 0.6–4.6)

**Notes:**

SD, Standard Deviation.

a Most common reason anxiety. One participant had received treatment for psychological ill-health before the child’s cancer.

b For the one child that had had a second cancer diagnosis, age at the second diagnosis is reported.

c For the one child that had had a second cancer diagnosis, second diagnosis is reported.

**Figure 1:**
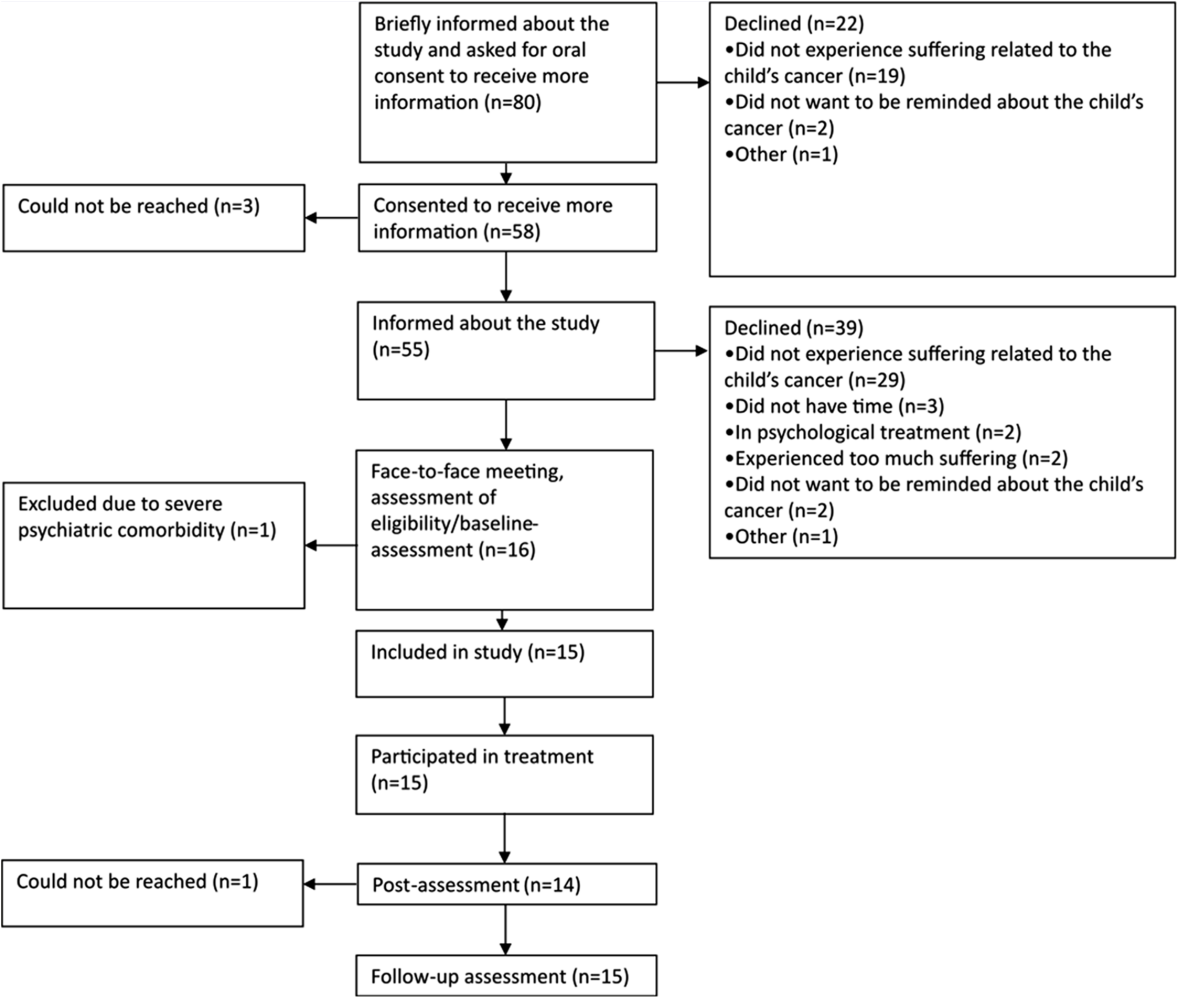
Participant flow through the trial.

Recruitment started 02/01/2013 and ended 02/15/2014. Potential participants were identified by staff at the at the pediatric oncology center at the Children’s University Hospital in Uppsala, who provided brief information about the purpose and procedures of the study. If a parent expressed interest to participate, and orally consented to receive more information, one of the psychologists working with the project contacted the parent via telephone to provide more information, to answer potential questions, and to assess inclusion and exclusion criteria. Parents who were judged eligible were scheduled for a face-to-face meeting with one of the psychologists working on the project. At the meeting, parents completed self-assessment forms, including questions about demographics (see Table 1), and were administered the M.I.N.I. Parents who fulfilled the inclusion criteria were offered participation, see Fig. 1. The same self-report measures and diagnostic interview were administered at the post-assessment (directly after completion of the intervention) and at the three-month follow-up assessment. Unstructured interviews were conducted before the start of treatment and the results from them will be reported elsewhere. The overall aim of these interviews was to identify the participants’ thoughts and feelings related to having a child previously treated for cancer. Interviews were conducted twice for each participant, and lasted until the participant had no further information to share. The interviews lasted approximately one hour each. Participants were not reimbursed for their participation in the study. The last follow-up assessment was conducted 12/04/2014. The mean time between the post-assessment and the follow-up assessment was 14 weeks (median 13.5 weeks).

### Psychological treatment

Due to the lack of knowledge regarding specific symptom topography and mechanisms involved in distress in this population, we chose not to develop a treatment manual beforehand, but instead chose to individualize the CBT to each participant’s needs. The individual face-to-face CBT was based on a behavioral case formulation approach to CBT in line with Persons (2012) and Sturmey (2008). The case formulation is an approach to individualize CBT by accounting for all the patient’s problems, disorders, and symptoms and to use learning theory to make explanatory inferences and generate hypotheses about the mechanisms causing and maintaining the patient’s problems (Persons, 2012; Tarrier, 2006). For this study, the behavioral case formulations were conducted for each participant during the first two to three sessions. The case formulations were discussed in the research group, including the two treating psychologists and three supervisors. The supervisors were licensed psychologists with clinical expertise in the patient group, and/or CBT development and evaluation, and/or behavior analysis. Each formulation encompassed a specification of the central problem/s as described by the participant, the topography was specified and functional analyses about its maintaining mechanism/s were generated (Dougher, 2000; Persons, 2012; Tarrier, 2006). Behavioral case formulations were discussed with each participant to ensure that their core problems were accurately addressed by the formulation. Based on the formulations, interventions were chosen to address the hypothesized maintaining mechanisms in accordance with previous literature describing best practice, i.e., ESTs, and drawing from applied learning theory, i.e., clinical behavior analysis and the concept of experiential avoidance (Dougher, 2000; Hayes et al., 1996). In short, such an approach is sensitive to the function of a problem behavior and aims to analyze the behavior in terms of reinforcement contingencies, i.e., under what condition the behavior occurs and what consequences it renders that may influence its frequency (Dougher, 2000). The concept of experiential avoidance suggests that attempts to avoid certain internal experiences (e.g., thoughts, feelings, memories, physical sensations) that are difficult to avoid (cf. ironic effects of thought suppression (Wegner, 1994)) or where the avoidance has other negative effects (e.g., difficulty functioning) is a core process underlying psychopathology (Hayes et al., 1996). See Table 2 for an overview of the intervention components used. See also Supplemental Information 1 for a more detailed specification of the intervention components.

**Table 2:** Intervention components used in the treatments and number of participants for whom the respective intervention was used.

Intervention components	Frequency *n* (%)
Psychoeducation	13 (87)
Functional analyses	12 (80)
Mindfulness	11 (73)
Behavioral activation	9 (60)
Exposure to cancer-related stimuli	8 (53)
General affect exposure	8 (53)
Relationship skills training	7 (47)
Defining values	6 (40)
Applied relaxation	4 (27)
Scheduling positive activities with the partner	4 (27)
Scheduling positive activities with the child	3 (20)
Targeting the worry process	3 (20)
Breathing training	2(13)
Exposure to health anxiety	2 (13)
Sleep hygiene	2 (13)
Anger management	1 (7)
Perfectionism exposure	1 (7)

**Notes:**

*n* = 15.

In addition to the intervention components mentioned above, general components such as setting goals for therapy and conducting a maintenance plan were included in treatments.

Two resident clinical psychologists conducted the treatments. After the unstructured interviews had been conducted, therapy sessions were given once a week and lasted for approximately 45 min. Parents were offered a maximum of 15 sessions and could use as many sessions as they preferred within this limit. The treatments took place in the pediatric oncology unit at the Children’s University Hospital, Uppsala or in the pediatric department at Västerås Hospital, depending on where the respective participant lived.

### Assessment of feasibility

To assess feasibility of the recruitment, the data collection and the treatment procedures, the number of potential and included participants, the reasons for not participating, the retention rates to treatment and data collection, the drop-out rates and the reasons for drop-out, were documented. Furthermore, duration of treatments, i.e., number and frequency of treatment sessions, and number of cancelled/re-scheduled sessions, were documented. Potential adverse effects were indicated by the number of participants reporting a higher level of psychological distress on the outcomes of primary interest after treatment completion. Also, potential associations between the outcomes of primary interest and the site of delivery of the intervention (Uppsala or Västerås), having the partner included in the study or not, and the time since the end of the child’s treatment, were calculated.

### Measurements

#### Outcomes of primary interest

Posttraumatic stress symptoms were assessed with the PTSD Checklist—Civilian Version (PCL-C) consisting of 17 items measuring symptoms of PTSD as defined in the B (re-experiencing), C (avoidance), and D (hyperactivity) criteria in DSM-IV (American Psychiatric Association, 1994). A value of 44 or above indicates a PTSD diagnosis (Blanchard et al., 1996). The PCL-C has shown good test–retest reliability and concurrent validity (Ruggiero et al., 2003; Weathers et al., 1993). In the current study, items were keyed to the child’s cancer disease. In prior research with this version including parents 12 months after the end of their child’s treatment for cancer, the mean (SD) was 28.7 (12.2) for mothers and 24.8 (8.9) for fathers (Ljungman et al., 2015). Cronbach’s α was 0.96 at baseline.

Anxiety was assessed with the Beck Anxiety Inventory (BAI; Beck et al., 1988) which consists of 21 items rated on a four-point scale ranging from never (0) to almost all the time (3), indicating how often the respondent had experienced anxiety symptoms. The BAI has shown high internal consistency (α = 0.94), good test–retest reliability (*r* = 0.67), and robust convergent validity (*r* = 0.54) (Fydrich, Dowdall & Chambless, 1992). Community norms indicate a mean (SD) of 6.6 (8.1) (Gillis, Haaga & Ford, 1995). Cronbach’s α was 0.93 at baseline.

Depression was assessed with the Montgomery–Åsberg Depression Rating Scale Self-assessment (MADRS-S: Svanborg & Åsberg, 1994), which consists of nine items measuring depressed mood over the past three days. The items consider areas such as appetite, concentration, mood, and sleep. Svanborg & Åsberg (2001) have reported that MADRS-S has good convergent validity with the Beck Depression Inventory (BDI) (*r* = 0.87) and suggested cut-offs are 0–12 no depression, 13–19 mild depression, 20–34 moderate depression, >34 severe depression. MADRS-S has good internal consistency and satisfactory test–retest reliability (Fantino & Moore, 2009). Cronbach’s α was 0.90 at baseline.

#### Outcomes of secondary interest

Worry was assessed with the Penn State Worry Questionnaire (PSWQ) which encompasses 16 items measuring excessive worry (Meyer et al., 1990). PSWQ has high internal consistency (Cronbach’s α = 0.91–0.95), good test–retest reliability (*r* = 0.92) (Meyer et al., 1990), and correlates highly with other questionnaires measuring anxiety and repetitive thinking (*r* = 0.67–0.73) (van Rijsoort, Emmelkamp & Vervaeke, 1999). Cronbach’s α was 0.93 at baseline.

Rumination was assessed with the rumination scale of the response style questionnaire (R-RSQ: Nolen-Hoeksema & Morrow, 1991). R-RSQ measures rumination as a response to symptoms of depression, consists of 22 statements, and has high internal consistency (Cronbach’s α = 0.89) (Nolen-Hoeksema, 1991) and good test–retest reliability (*r* = 0.67) (Treynor, Gonzalez & Nolen-Hoeksema, 2003). Cronbach’s α was 0.91 at baseline.

Experiential avoidance was assessed with the Acceptance and Action Questionnaire-II (AAQ-II; Bond et al., 2011). AAQ-II in its original form consists of 10 items measuring experiential avoidance. The instrument has good internal consistency and test–retest reliability (Bond et al., 2011). The convergent validity of the AAQ-II is good; it correlates significantly positive with measures of depression, anxiety, and thought suppression (Bond et al., 2011). In the present study, the items were cued to the child’s cancer, and six extra items measuring avoidance of cancer-related experiences were included (Cernvall et al., 2013). Cronbach’s α was 0.72 at baseline.

Quality of life was assessed with the Satisfaction with Life Scale, which consists of five items where the individual is asked to compare the current situation with a hypothetical standard (e.g., “I am satisfied with my life”; Diener et al., 1985). Individuals rate the statements on a seven-point scale from 1 = strongly disagree to 7 = strongly agree. The instrument has good test–retest reliability (*r* = 0.82), high internal consistency (Cronbach’s α = 0.87) (Diener et al., 1985), and adequate convergence with related measures (Pavot & Diener, 1993). Cronbach’s α was 0.85 at baseline.

A psychologist administered the M.I.N.I. structured diagnostic psychiatric interview for DSM-IV and ICD-10 to assess psychiatric disorders (Sheehan et al., 1998). At baseline, the psychologist who worked as the patient’s therapist administered the diagnostic interview and data collection; at the following assessments another psychologist not familiar with the participant administered the diagnostic interview and the data collection. On a few occasions, the treating psychologist collected the data (three occasions during post-assessment and one occasion at a follow-up assessment) for administrative reasons.

### Statistical analyses

Statistical analyses were performed using IBM SPSS 22.0. Potential changes from baseline to post-assessment, and from baseline to follow-up assessment, were analyzed using a dependent *t*-test. Within-group effect sizes were estimated using Cohen’s *d* based on baseline to post-assessment and baseline to follow-up assessment change scores. According to Cohen (Cohen, 1988), effect sizes of *d* = 0.2, *d* = 0.5, and *d* = 0.8 are considered small, medium, and large, respectively. For the outcomes of primary interest (PCL-C, BAI, MADRS-S) the proportion of study participants who met the criteria for reliable change according to Jacobson & Truax (1991) was calculated. This approach assesses whether the magnitude of change for each individual, in relation to the test–retest reliability of the measurement, is beyond what could be expected by chance. Potential differences and/or associations between the outcomes of primary interest and the site of delivery of the intervention, having the partner included in the study or not, and time since end of the child’s treatment were calculated using independent *t*-test or Pearson correlation.

The few missing items (one item in AAQ-II and one item in R-RSQ at baseline; two items in AAQ-II at post-assessment; and one item in AAQ-II at follow-up assessment) were imputed using the average item score on the respective measurement for the respective individual at that assessment point. As one participant did not complete post-assessment, list-wise deletion was used for the post-treatment analyses.

### Cognitive behavioral conceptualization

In order to derive a general conceptual model, the individual behavioral case formulations were aggregated according to the following procedure: Continuous discussions were held during the course of the study between the three supervisors and the two psychologists working as therapists in the study. The general conceptualization gradually evolved during the course of the study. The conceptualization was guided by principles as outlined in behavioral case formulation approaches (Persons, 2012; Sturmey, 2008) and was influenced by contemporary models of depression (Martell, Addis & Jacobson, 2001), PTSD (Foa & Kozak, 1986), and the concept of experiential avoidance (Hayes et al., 1996). A summarizing meeting where all case formulations were read and discussed by the psychologists working with the treatments and the supervisors was held to assure that the conceptualization was representative for the case formulations. After completion of the study, all documentation about each patient, including the behavioral case formulations, the treatment summary and all patient journal data, were carefully re-read by one of the first authors (LL) and once again summarized with regard to each patient’s presenting problems and all interventions used in the treatments, to ensure that no relevant information had been omitted in the case formulation or the analyses of the material. This was done by extraction and summation of “central problems,” “hypothesized maintenance mechanisms,” “interventions,” “homework assignments,” and “material provided to participant” from each treatment summary. Types and frequencies were compared to the general conceptualization. As such, the goal was to generate a framework informed by CBT theory describing psychological distress and hypothesized maintenance mechanisms.

**Figure 2:**
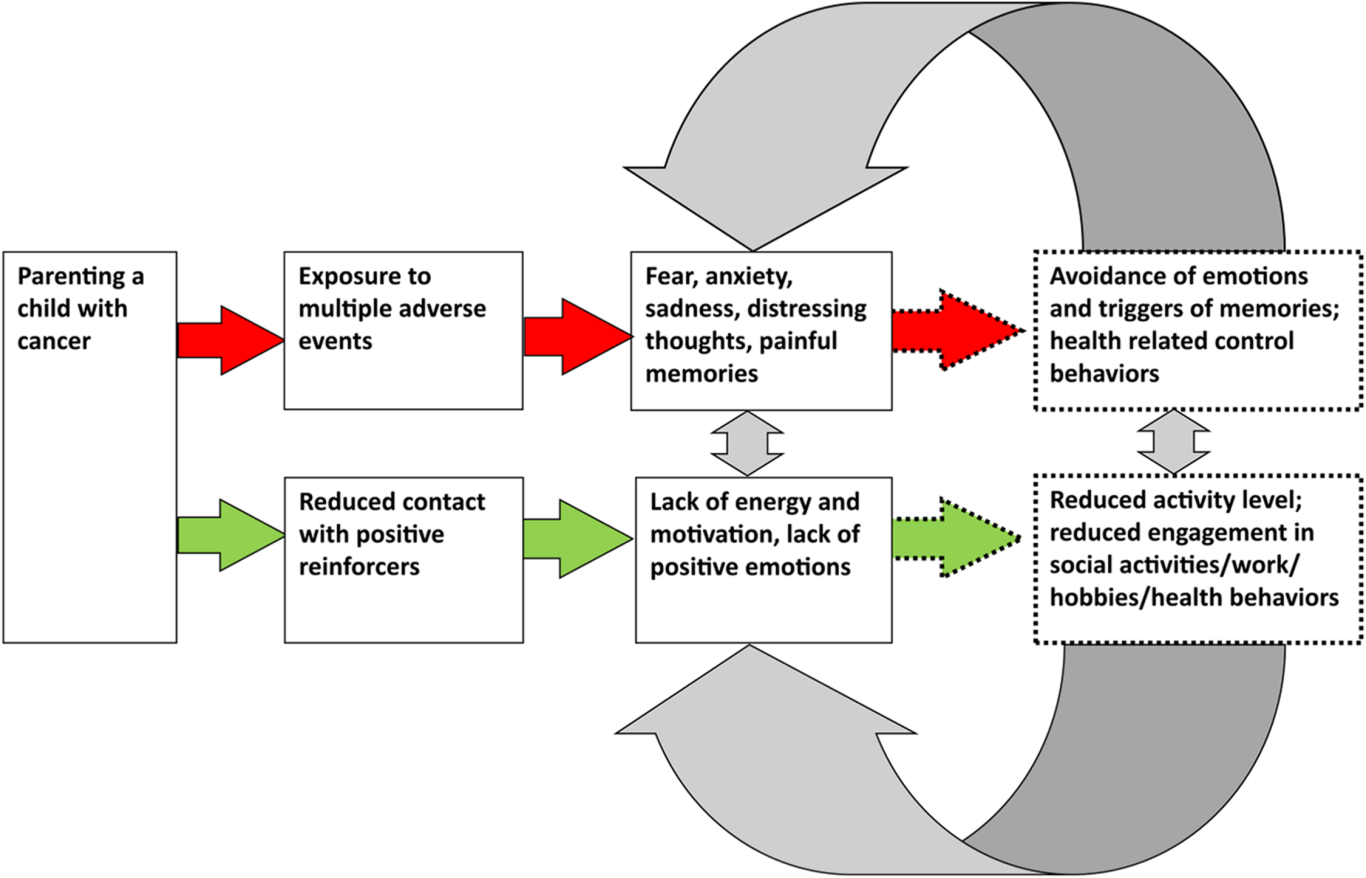
Development and maintenance of symptoms of traumatic stress and of depressive symptoms.

The drafted conceptualization was validated via a participatory action research (PAR) approach (Kindon, Pain & Kesby, 2007). Four of the participants in the current study and two participants in a previous intervention study by our group (Cernvall et al., 2015) participated as parent research partners (PRPs) in the PAR study. The overall aim of the PAR study was to transform the findings of the present study to an internet-administered, guided, self-help intervention (Wikman et al., in press). The PRPs gave feedback on the general conceptualization (depicted in Fig. 2) in terms of the categorization of psychological distress as symptoms of traumatic stress and depressive symptoms, and the hypotheses regarding developmental and maintenance factors.

## Results

### Feasibility

Of the 80 potential participants, 15 were included, representing a 19% inclusion rate. Of those who declined participation, 48 (60%) reported “not experiencing suffering related to the child’s cancer” as the reason for declining participation. Consequently, these did not belong to the target population. Of the 32 remaining potential participants, the 15 included participants represented an inclusion rate of 47% of the target population. The most common reason to decline participation among the remaining potential participants was “Do not want to be reminded about the child’s cancer” (mentioned by four parents). Figure 1 shows the participant flow through the trial, including reasons for not participating.

All but one of the 15 participants completed the treatment, representing a treatment retention rate of 93%. One participant withdrew from treatment after five sessions due to time constraints and could not be reached for collection of post-assessment data. The follow-up assessment was completed by all 15 participants. Treatments lasted between two and 22 weeks, with a mean of 13.3 weeks and comprised 2–15 sessions (Mean = 10 sessions, SD = 4.7, Median = 12 sessions). At mean, 0.8 sessions were held per week and the mean number of cancelled/re-scheduled sessions per participants was 1.0.

At post-assessment, one participant reported an increase by four points on the PCL-C, and one participant reported an increase by one point on the MADRS-S compared to baseline. At follow-up, two participants reported an increase by two points on the BAI compared to baseline. Additionally, one participant reported no change on the BAI between baseline and post-assessment and two participants reported no change on the MADRS-S between baseline and follow-up. At follow-up, all participants reported reductions on the PCL-C when compared to baseline.

There were no indications of a relationship between site of delivery (Uppsala vs. Västerås) and level of reported distress in the outcomes of primary interest at any time point (*p* = 0.06–0.97), change in these outcomes at post-assessment or follow-up (*p* = 0.32–0.69), and/or to number of treatment sessions (*p* = 0.81). Similarly, having the partner included in the study or not was not related to reported distress in the outcomes of primary interest at any time-point (*p* = 0.15–0.93), nor to a change in them at post-assessment or follow-up (*p* = 0.07–0.99), and/or the number of sessions (*p* = 0.72). Lastly, time since end of the child’s treatment was not related to the outcomes of primary interest at baseline (*p* = 0.13–0.20), nor to any change in them at post-assessment or follow-up (*p* = 0.17–0.67).

### Change in the outcomes

Results from the self-reported outcomes are summarized in Table 3. Significant improvements in PTSS, symptoms of depression, symptoms of anxiety, and experiential avoidance were seen from baseline to post-assessment. From baseline to follow-up assessment, participants reported significant improvements on all outcomes. For the outcomes of primary interest (PTSS, symptoms of depression, and symptoms of anxiety) reductions from baseline to post-assessment, and from baseline to follow-up assessment were all significant at least at the level of *p* < 0.01. Effect sizes were in the medium-to-large range, see Table 3. For PTSS and symptoms of depression, effect sizes at the follow-up assessment were large (*d* = 1.30 and *d* = 1.28 respectively).

**Table 3:** Baseline, post-, follow-up assessment, differences, and effect sizes for all outcome measures.

	Mean (SD)						
	Baseline (*n* = 15)	Post (*n* = 14)	Follow-up (*n* = 15)	*T* Baseline/Post (*n* = 14)	*t* Baseline/Follow-up (*n* = 15)	*d* Baseline/Post [95% CI] (*n* = 14)	*d* Baseline/Follow-up [95% CI] (*n* = 15)
PCL-C	49.3	34.0	28.7	5.1***	5.8***	0.92	1.30
	(17.6)	(14.6)	(10.1)			[0.49, 1.35]	[0.65, 1.96]
BAI	16.6	8.7	8.6	4.2**	3.2**	0.65	0.69
	(12.5)	(10.3)	(6.8)			[0.33, 0.97]	[0.18, 1.20]
MADRS-S	18.8	11.0	8.3	4.8***	5.1***	0.85	1.28
	(9.3)	(6.1)	(5.5)			[0.43, 1.26]	[0.55, 2.01]
PSWQ	50.2	41.5	38.4	1.6	3.2**	0.66	0.94
	(14.6)	(11.6)	(9.4)			[−0.17, 1.50]	[0.18, 1.70]
R-RSQ	43.9	37.9	36.8	2.0	2.7*	0.58	0.73
	(10.2)	(10.3)	(9.1)			[0.01, 1.15]	[0.08, 1.37]
AAQ-II	51.4	39.3	36.7	2.8*	4.7***	0.84	1.08
	(13.4)	(15.2)	(13.7)			[0.20, 1.48]	[0.46, 1.70]
SWLS	21.9	25.0	25.5	−2.1	−2.6*	−0.52	−0.62
	(6.4)	(4.3)	(4.5)			[−0.97, −0.07]	[−1.18, −0.05]

**Notes:**

PCL-C, The PTSD Checklist-Civilian Version; BAI, Beck Anxiety Inventory; MADRS-S, Montgomery–Åsberg Depression Rating Scale Self-assessment; PSWQ, Penn State Worry Questionnaire; R-RSQ, Rumination Scale of the Response Style Questionnaire; AAQ-II, Acceptance and Action Questionnaire-II; SWLS, Satisfaction with Life Scale; SD, Standard Deviation; *t*, dependent *t* test statistics for potential differences between assessments; *d*, Cohen’s *d* for effect sizes; CI, Confidence Interval.

* *p* < 0.05.

** *p* < 0.01.

*** *p* < 0.001.

The proportions of participants who met criteria for reliable change on the outcomes of primary interest are reported in Table 4. At follow-up, the proportions ranged from 33% to 87%, with the highest proportion for PTSS. The results from the clinical diagnostic interviews are reported in Table 5. At baseline, eight participants (53%) fulfilled the criteria for at least one diagnosis, compared to three (20%) at post-assessment and three (20%) at follow-up assessment. Major depressive disorder was the most common diagnosis at baseline (seven participants), followed by PTSD (four participants). At post- and follow-up assessment, no participant fulfilled the criteria for neither major depressive disorder nor PTSD.

**Table 4:** Reliable change from baseline to post-assessment and from baseline to follow-up assessment.

	Baseline/Post (*n* = 14)	Baseline/Follow-up (*n* = 15)
PCL-C *n* (%)	10 (71.4)	13 (86.7)
MADRS-S *n* (%)	4 (28.6)	8 (53.3)
BAI *n* (%)	3 (21.4)	5 (33.3)

**Notes:**

Reliable change scores were 9.7 for PCL-C, 9.3 for MADRS-S, and 9.2 for BAI.

PCL-C, The PTSD Checklist-Civilian Version; BAI, Beck Anxiety Inventory; MADRS-S, Montgomery–Åsberg Depression Rating Scale Self-assessment.

**Table 5:** Number of individuals meeting the criteria for psychiatric disorders according to the M.I.N.I. structured diagnostic psychiatric interview.

	Baseline (*n* = 15)	Post (*n* = 14)	Follow-up (*n* = 15)
Major depressive disorder	7	0	0
Dysthymia	1	2	1
Non-severe suicidal ideation	2	0	0
Panic disorder	2	0	0
Obsessive compulsive disorder	2	0	1
Posttraumatic stress disorder	4	0	0
Alcohol dependency/addiction	0	1	1
Substance dependency (non-alcohol)	1	0	0
Generalized anxiety disorder	3	1	1
At least one diagnosis	8	3	3
Two diagnoses	3	1	1
More than two diagnoses	4	0	0

**Note:**

Two participants meeting the criteria for posttraumatic stress disorder related this to another trauma besides the child’s cancer.

### Cognitive behavioral conceptualization

The aggregated behavioral case formulations resulted in a conceptualization consisting of two parallel but overlapping paths describing development and hypothesized maintenance of symptoms of traumatic stress and of depressive symptoms (see Fig. 2). The model draws heavily on previous CBT models for psychological distress, in particular the PTSD-model described by Foa & Kozak (1986; McLean & Foa, 2011), the depression-model described by Martell, Addis & Jacobson (2001; Martell, Dimidjian & Herman-Dunn, 2010), and the concept of experiential avoidance described by Hayes et al. (1996).

In the model, the boxes on the left side describe the psychologically relevant experiences involved in parenting a child under treatment for cancer, and the emotional and cognitive responses to be expected when exposed to them. The boxes on the right-hand side of the model (dashed lines) illustrate the behaviors hypothesized as core maintaining processes, i.e., behaviors involved in the development and maintenance of symptoms of psychological distress. Importantly, we hypothesize that the maintenance behaviors have evolved through the parents’ adaptation to the context of the child being ill and under treatment for cancer since these behaviors, in that specific context, likely have been helpful to parents, e.g., in order to manage practical issues, and to cope with difficult emotions and cognitions associated with the child’s illness. We call these state of emergency behaviors (SEBs). We now turn to a specification of the two hypothesized pathways, and the SEBs involved in them.

#### Traumatic stress-pathway

Symptoms of traumatic stress included painful memories from the time of the child’s illness, emotional numbness, health-related control behaviors, future-oriented worry, and symptoms of hyperarousal such as concentration and sleep difficulties. Additionally, formal flashbacks and nightmares were reported. We assume that these symptoms stem from the multiple and repeated adverse events that parents have been exposed to during the time of the child’s illness. When exposed to such experiences, emotional and cognitive responses, e.g., fear, anxiety, sadness, distressing thoughts, and painful memories likely arise (left-hand side of the model, see Fig. 2). According to this model, behavioral responses in the context of having a child under treatment for cancer and that may serve the function of SEBs, are: high degree of focus on, and controlling of, symptoms of disease in the child, avoidance of situations that involve risk of infectious diseases, a shift of focus towards managing disease and threat, and use of emotion-controlling strategies such as emotion and thought suppression. We hypothesize that these behaviors serve adaptive functions for the time of the child’s illness such as helping to keep the child free from potentially lethal infections (health-related control behaviors), helping the parents to cope with the experience of, e.g., forcibly holding one’s child during painful medical procedures (distraction from one’s own emotional reactions), and helping parents to handle the overall situation involving the threat to the child’s life (avoidance of painful thoughts and emotions). However, through operant learning, primarily via negative reinforcement, we hypothesize that some parents continue to engage in the same or similar SEBs even after the end of the child’s treatment. In this context, the SEBs will no longer serve adaptive functions, but will rather be related to maladaptive adjustment, development and maintenance of symptoms of traumatic stress (see Fig. 2 on the right-hand side, the arrows and boxes with dotted lines). As an example, after completion of the child’s cancer treatment, emotional avoidance has no beneficial effects such as managing adverse situations, but instead hinders processing of distressing memories and emotions related to the cancer experience, and thus maintains feelings such as fear and sadness. It will also potentially lead to secondary negative effects (e.g., experience of alienation and relationship difficulties). The continuous use of disease-managing behaviors such as a high degree of health-related control behaviors and continuous focus on threat maintains fear and the experience of being under immediate threat. Also, future-oriented worry and concerns will likely serve fewer adaptive functions when the child is no longer ill and under treatment since parents no longer need to be emotionally and practically prepared for the repeated difficult situations associated with having a child undergoing treatment for cancer. Thus, the use of an inflexible behavior repertoire stemming from the time of the child’s illness is hypothesized as a core feature of the development and maintenance of traumatic stress in this population. It is also important to highlight that the parents frequently reported worry and anxiety related to late medical effects and a potential future relapse, which are realistic and current stressors that parents of children previously treated for cancer face. Given this, remaining worry and concerns about late effects are also therefore likely to be expected for parents who have adapted well to the situation. Still, we hypothesize that the use of SEBs likely interferes with the adaptation to these remaining stressors, and hinders adaptive coping with the child’s late effects, and the potential risk of a future relapse.

#### Depression-pathway

The depressive symptoms mainly included lack of energy and motivation, and lack of positive emotions. These symptoms were hypothesized to stem from the prolonged reduced access to positive reinforcements during the time of the child’s illness. This involves not being able to live in one’s own home, to work, and importantly, to spend time with the family and the partner due to the long hospital stays and the sick child’s extensive care needs. The expected reaction during such circumstances is a lowered level of energy and motivation, as well as a lowered level of positive emotions (Martell, Addis & Jacobson, 2001). As for traumatic stress, we hypothesize that there are SEBs relevant to the development of depressive symptoms, i.e., behaviors that have been established through the adaptation to the child’s illness. Examples of SEBs involved in the development and maintenance of depressive symptoms are: a shift of focus towards prioritizing to manage the child’s disease, decreased engagement in social life and work-related tasks, reduced time spent on physical exercise, hobbies, and everyday household tasks, and reduced planning for the future. We hypothesize that these behaviors have served adaptive functions during the time of the child’s illness and have been helpful to parents in order to manage the extraordinary situation of parenting a child on cancer treatment. By down-prioritizing one’s own needs, full focus can be given to the child and to managing the demanding treatment regimens. However, when the child’s treatment is completed, some parents continue to use these SEBs, and will thereby maintain a low degree of contact with potential positive reinforcers, and thus a low frequency of positive emotions and perceived energy level and motivation (Martell, Addis & Jacobson, 2001) (Fig. 2 on the right-hand side, the arrows and boxes with dotted lines). This vicious circle of depressive symptoms and behavioral inactivity is well described in the literature (Martell, Dimidjian & Herman-Dunn, 2010). However, the establishment of low levels of activities through the adaptation to the child’s illness period is hypothesized as a unique feature for the development of depressive symptoms in the context of parenting a child with cancer.

#### Overlap between the pathways

We argue that there is an intricate overlap between the two pathways leading to symptoms of traumatic stress and depression. This overlap is suggested to be mediated by SEBs feeding into both pathways. One such SEB is emotional avoidance described above in relation to symptoms of traumatic stress. Emotional avoidance will also contribute to the development and maintenance of depressive symptoms if this behavioral strategy is used excessively as it will imply low engagement/presence in activities, and thereby hinder access to potentially positive reinforcers (Ellard et al., 2010). Interestingly, several participants reported a rather high level of participation in activities that used to be reinforcing to them, however, the functional analyses revealed that they had low contact with the present moment during these activities and approached them in a rather numbed state. Furthermore, the use of health-related control behaviors (identified as SEBs in the traumatic stress pathway), such as avoiding social events to avoid infections, will maintain symptoms of depression as well as it implies a lowered level of participation in activities. Likewise, internal health-related control behaviors such as worrying about disease will diminish contact with the present moment during activities, and thus maintain symptoms of depression. Also, low engagement in activities, hobbies, etc., identified as an SEB involved in the depressive pathway, will potentially maintain symptoms of traumatic stress. This could occur when low engagement in activities reduces opportunities for natural exposure to triggers of emotions related to the child’s cancer disease. An example of this is reduced contact with friends, which both leads to a lack of potential positive reinforcement, and to lost opportunities to talk about difficult experiences related to the child’s cancer.

In the respondent validation in the PAR study (Wikman et al., in press), the PRPs overall considered the model to be relevant and representative of their experiences and coherent with their perceptions of the maintaining mechanisms that had been targeted by the interventions in the CBT that they had participated in. They provided suggestions regarding the wording when communicating the model to future participants. For example, the PRPs preferred the term “changed life situation” rather than “depressive inactivity,” and preferred “difficult/painful emotions and memories” rather than “traumatic stress.” These more technical concepts have been kept in this scientific report and presentation of the conceptual model, but the more layperson-oriented wording is used when communicating the model to representatives of the population.

## Discussion

This study is, to the best of our knowledge, the first preliminary evaluation of individualized face-to-face CBT for parents of children after end of treatment for childhood cancer. It is also the first study to present a cognitive behavioral conceptualization of the distress experienced by these parents. The treatment and the study procedures appeared feasible and acceptable to participants. During the course of the trial, participants reported significant improvements in all outcomes of primary interest (PTSS, symptoms of depression, and symptoms of anxiety). At the three-month follow-up, participants reported significant improvements in all outcome variables. Effect sizes for PTSS, symptoms of depression, and symptoms of anxiety were medium to large at post- and follow-up assessments. The largest effects were seen for PTSS and symptoms of depression at follow-up. At follow-up, 87% reported a reliable improvement in PTSS; the corresponding figures for symptoms of depression and anxiety were 53% and 33%, respectively. Overall, participants reported improvements in several self- as well as clinician-assessed aspects of psychological distress after the end of treatment.

The psychological intervention evaluated in the current study was based on a behavioral case formulation approach where functional analyses were conducted for each participant’s specific problems. This approach has been suggested for psychological problems where no psychiatric diagnosis is manifested, or when patients report comorbid psychiatric diagnoses, or diffuse or sub-clinical levels of psychiatric syndromes (Persons, 2012). The aggregated behavioral case formulations resulted in a conceptualization of cancer-related psychological distress in parents of children after end of treatment for childhood cancer. We found that the conceptualization and treatment of PTSD (Foa & Kozak, 1986) and of depression (Martell, Addis & Jacobson, 2001) fit well with the current population, and our conceptualization could be viewed as an integration of the guiding principles in these two conceptualizations. Thus, our conceptualization consists of two separate yet overlapping pathways; the traumatic stress pathway and the depressive symptoms pathway, and included hypotheses on development and maintenance of these symptoms. In line with the previous conceptualizations, we hypothesize avoidance of cancer-related stimuli (including external stimuli and internal stimuli such as thoughts and emotions related to the cancer experience) and low engagement in potentially reinforcing activities to be core maintaining mechanisms of the distress. The cognitive and behavioral techniques utilized in the current study (presented in Table 2) were not invented by us, but are well established in the field (see Supplemental Information 1). However, we further hypothesize that the suggested mechanisms are a consequence of the adaptation to the challenging circumstances at the time of the child’s illness, and use the term SEBs to describe these behaviors. This is a novel contribution to this field and even though this conceptualization is merely a hypothesis and in need of further research for validation there is research giving some support to the importance of similar caregiver behaviors during the time of the child’s illness and treatment in the development of psychological distress. For example, Norberg, Lindblad & Boman (2005) showed that avoidance was not associated with psychological distress among parents of children 1–8 weeks after their child’s cancer diagnosis. However, avoidance was associated with psychological distress 5–10 years after diagnosis, suggesting that avoidance may not be problematic when used shortly after diagnosis, but later in the disease trajectory. In our hypothesized conceptualization, future-oriented worry is incorporated in the traumatic stress pathway. To some extent, worry in this population can be viewed as related to the ongoing risk of cancer recurrence. This is a realistic aspect of the situation parents face, and a risk that they have to cope with. Still, we believe that excessive worry can be considered an SEB related to the repeated, distressing, and demanding potentially traumatic events occurring during the time of the child´s illness. However, after end of the child’s treatment, continuous preparation for recurrence may instead serve maladaptive functions and maintain symptoms of traumatic stress. Worry may therefore be a symptom that occurs more frequently in relation to the current type of traumatic event (i.e., being a parent of a child after the end of treatment) compared to more past-oriented and time-discrete traumatic events. However, we believe that the future-oriented worry that the parents report fits within the concept of traumatic stress, both as a symptom and with regard to development and maintenance.

Several studies have highlighted that the symptoms reported by the present population do not fit into the current nomenclature for psychiatric disorders, and that there is no conceptualization of the distress upon which to base an intervention (Bruce, 2006; Cernvall et al., 2013; Ljungman et al., 2014). The behavioral case formulation approach allowed for tailoring intervention strategies, as well as gathering information about the symptoms of distress and the mechanisms involved in the development and maintenance of them, in the current population. One way of validating the behavioral case formulations is to evaluate the impact of the chosen interventions on the symptoms addressed (Persons, 2012). In line with the hypotheses generated in our case formulations, the impact from the treatment seemed to be most evident for symptoms of traumatic stress and symptoms of depression, providing some validity to the case formulations and the hypotheses regarding the maintenance of mechanisms.

The overall inclusion rate, 19%, roughly corresponds with the expected prevalence of psychological distress in the population (Ljungman et al., 2014, 2015). The inclusion rate calculated without the potential participants that reported “not experiencing psychological suffering,” and thereby did not belong to the target population, was 47%. Several of the previous attempts to intervene in the current population have encountered difficulties with regard to recruitment (Cernvall et al., 2015; Stehl et al., 2009). We did not encounter such difficulties in the present study. One important reason for this could be that the intervention was offered to parents after successful completion of the child’s treatment. The high recruitment rate indicates that the period after end of the child’s treatment might be the right time to offer psychological interventions to parents of children diagnosed with cancer. Furthermore, the retention rate to treatment was high; 93% of the parents completed the treatment. This finding could be related to study participation only being offered to parents reporting psychological distress, and thereby resulting in participants highly motivated to treatment. The flexibility in treatment length and content to participants’ individual needs could further explain the high retention rate. Given the exploratory nature of this study, we did not have any pre-conception of what would constitute an ideal number of sessions for optimal outcome, although a maximum of 15 sessions was set. Despite this, participants received a median of 12 sessions, which corresponds quite well with previous research on behavioral activation (Cuijpers, Van Straten & Warmerdam, 2007) and treatment for PTSD (Powers et al., 2010), and with research on what constitutes a sufficient dose of psychological intervention. For example, in a review of the literature, Hansen, Lambert & Forman (2002) found that 67% of participants achieved reliable change within an average of 12.7 sessions. Also, sessions were scheduled independently of any medical appointments the child may have had at the hospital. The feasibility of this procedure is unknown to us due to lack of systematic data. It remains an empirical question for future studies to investigate whether scheduling intervention sessions next to, or close to medical appointments would be perceived as more feasible, desirable, or counterproductive. Overall, the recruitment, data collection, and administration of psychological treatment were conducted with relative ease, indicating that the study procedures and the psychological treatment offered was acceptable and engaging for the participants. Importantly, there were few indications of worsening of symptoms after having completed treatment, suggesting that the interventions is safe for this group of parents.

Some limitations should be addressed. First, as an in-depth understanding of the psychological distress experienced by the population at hand has been lacking, an a priori decision of a single primary outcome was not possible. We chose PTSS, symptoms of depression, and symptoms of anxiety as outcomes of primary interest and based on our case formulations, symptoms of traumatic stress and depression were the primary symptoms addressed in treatment, which is also reflected in the resulting conceptualization. However, it is important to stress that the case formulations, and the resulting conceptualization as well as the mechanisms of distress identified, are merely our hypotheses. Potentially different ways to interpret and conceptualize distress in this population should be considered. It is up to future studies to further explore the symptom topography and the mechanisms involved in the distress in this population, and to determine the most relevant outcomes. Furthermore, as we did not use a standardized treatment manual, we chose not to perform any formal assessment of therapist adherence. However, each therapist carefully documented all intervention techniques used in order to increase transparency of treatment content. Moreover, we did not conduct any formal assessment of treatment acceptability or treatment satisfaction. However, the low drop-out rate may be viewed as an indicator of patient acceptability. Furthermore, we did not include any measurement regarding the well-being and/or psychological distress experienced by the participants’ children. Potential downstream effects of the intervention on the children, such as decreased psychological distress, should be considered in forthcoming studies. Lastly, the uncontrolled design of this study does not allow conclusions regarding treatment efficacy. It may be the case that improvements observed in this study may be due to other factors than the intervention provided. It is important that future studies use designs that allow for inferences regarding the efficacy of the treatment.

## Conclusion

The development and preliminary evaluation of a psychological intervention for parents of children after the end of treatment for childhood cancer and the resulting conceptualization of distress responds to a gap in the clinical care of this population as well as the theoretical understanding of their distress. Results from the current study suggest that the psychological treatment was feasible and acceptable, and significant reductions of distress during the intervention were observed. Furthermore, individual case formulations were aggregated and presented as a cognitive behavioral conceptualization of distress among these parents, including traumatic stress symptoms and depressive symptoms and their hypothesized maintenance mechanisms. As the next step in the process of developing a treatment for this population, we have integrated this conceptualization and the interventions used in the current study into a guided and internet-administered self-help intervention (Wikman et al., in press) which will be tested in a forthcoming Phase II feasibility study. If shown to be acceptable and feasible, its efficacy will be evaluated in a controlled trial. If the intervention is proven to be efficacious, it could be provided to the population via pediatric oncology follow-up, and/or late-effects units or via relevant organizations.

## Supplemental Information

10.7717/peerj.4570/supp-1 Supplemental Information 1 Appendix: Specification of interventions used in the treatments.

10.7717/peerj.4570/supp-2 Supplemental Information 2 TREND checklist.

10.7717/peerj.4570/supp-3 Supplemental Information 3 Trial protocol: version approved by the regional ethical vetting board.

## Additional Information and Declarations

### Clinical Trial Registration

The following information was supplied regarding Clinical Trial registration: ISRCTN74785895.

### Data Availability

The following information was supplied regarding data availability: Ethical restrictions prohibit us from making the dataset publicly available. The ethical approval obtained ensures use of the dataset to answer the stipulated research questions and to ensure that the data are processed in accordance with the Swedish Personal Data Act. Data are available at request from the research group Clinical Psychology in Healthcare at the Department of Women´s and Children´s Health, Uppsala University for researchers who meet the criteria for access to confidential data. Requests should be sent to PI Prof. Louise von Essen, louise-von.essen@kbh.uu.se.
