# Supplementary material for: An open trial of individualized face-to-face cognitive behavior therapy for psychological distress in parents of children after end of treatment for childhood cancer including a cognitive behavioral conceptualization
Source: PeerJ. 2018 Apr 11;6:e4570. doi: 10.7717/peerj.4570 (PMC5899418; doi:10.7717/peerj.4570)
Supplement: Supplemental Information 1 [file peerj-06-4570-s001.docx]

**S1 Appendix.** **Specification of interventions used in the treatments.**

Figures in parentheses indicate number of participants for whom the respective intervention was used. A total of 15 participants were included in the study.

*Psychoeducation (n=13, 87%)*

Psychoeducation was provided by the psychologist during sessions and as homework using written material or at a few occasions as audio files. Psychoeducation included information about stress and anxiety, the consequences of avoidance (Barlow, 2008; Hayes, Wilson, Gifford, Follette, & Strosahl, 1996), depressive inactivity (Martell, Addis, & Jacobson, 2001), parenting skills (Forster, 2009), health anxiety (Hedman et al., 2011), posttraumatic stress disorder (Foa, Hembree, & Rothbaum, 2007), mindfulness, defusion and acceptance (Cernvall, Carlbring, Ljungman, & von Essen, 2013; Hayes et al., 1996), applied relaxation (Öst, 1987), exposure techniques (Craske, Treanor, Conway, Zbozinek, & Vervliet, 2014), and worry and rumination (Martell, Dimidjian, & Herman-Dunn, 2010) tailored to each participant’s needs.

*Functional analyses (n=12, 80%)*

Functional analyses were conducted during sessions and as homework. Participants were trained in applying such analyses on their own problematic behaviors by noticing and analyzing antecedents, the emotional, physical and cognitive reactions, the behavioral responses, and the short- and long-term consequences of these (Persons, 2012; Sturmey, 2008).

*Mindfulness (n=11, 73%)*

Mindfulness exercises were conducted together with the psychologist during sessions and as homework (Baer, 2006; Hofmann, Sawyer, Witt, & Oh, 2010). The exercises focused on internal stimuli such as emotional reactions, physical sensations, and cognitive processes. Participants were trained in adopting an open and accepting approach towards internal stimuli.

*Behavioral activation (n=9, 60%)*

Behavioral activation (Martell et al., 2001, 2010) included registration of daily activities and the association between these and mood, identification of potentially reinforcing activities, and scheduling of such activities.

*Exposure to cancer-related stimuli (n=8, 53%)*

Exposure to cancer-related stimuli included e.g., conversations about experiences related to the child’s cancer (during sessions and as homework with family and friends), reading diaries from the time of the child’s illness (for participants who had written such), watch documentaries about cancer, observe cancer related scarring on the child’s body, and eat food associated to the child’s illness period (Craske et al., 2014; Foa, Keane, Friedman, & Cohen, n.d.). Additionally expressive writing assignments were used (Baikie & Wilhelm, 2005; Pennebaker, 1997).

*General affect exposure (n=8, 53%)*

General affect exposure included exercises to increase awareness of emotional responses in general and to verbalize and communicate these (in sessions and as homework with e.g., the partner). During sessions participants were asked to recall situations that had triggered emotional responses, and were guided to take an observing and accepting approach towards potential reactions. As homework assignments participants used “emotion time”; a specific time during the day when the participant was instructed to direct awareness towards present emotional reactions and recall previous emotional reactions using mindfulness techniques. Participants wrote diaries focusing on emotional reactions and used mindfulness techniques as homework to increase attention to emotional reactions (Chambers, Gullone, & Allen, 2009; Hayes et al., 1996; Hofmann et al., 2010).

*Relationship skills training (n=7, 47%)*

Relationship skills training included practicing skills in communicating during sessions and as homework. Exercises included communicating own needs, giving positive feedback, and scheduling time with partner once a week to discuss practical and emotional issues (Hawkins, Blanchard, Baldwin, & Fawcett, 2008).

*Defining values (n=6, 40%)*

Defining values included discussing important life values during sessions, homework assignments such as mapping central areas in life and identify valued direction in these, and identifying values related to being a parent (Hayes, Strosahl, & Wilson, 1999).

*Applied relaxation (n=4, 27%)*

Applied relaxation was mainly used as homework assignments and included written instructions and audio files (Öst, 1987).

*Scheduling positive activities with the partner (n=4, 27%)*

Scheduling positive activities with the partner included identifying positively reinforced activities that can be performed together with the partner, and to schedule and perform such with the partner (Hawkins et al., 2008).

*Scheduling positive activities with the child (n=3, 20%)*

Scheduling positive activities with the child included identifying positively reinforced activities that can be performed together with the child, and to schedule and perform such with the child (Forster, 2009).

*Targeting the worry process (n=3, 20%)*

Targeting the worry process was done by teaching the participant to identify and terminate worry behaviors (internal and external), postpone worry to a specific scheduled time later during day when subject of worry is fully thought through (Borkovec & Costello, 1993), and by applying an acceptance- and willingness perspective to the content of thoughts (Hayes et al., 1999).

*Breathing training (n=2, 13%)*

Breathing training was conducted according to the prolonged exposure manual (Foa et al., 2007).

*Exposure to health anxiety (n=2, 13%)*

Exposure to health anxiety included exposure with response prevention to stimuli eliciting health anxiety related to own health issues (Hedman et al., 2010) such as to book an appointment with a physician to get a health examination, or directing awareness into the body by using interoceptive exposure techniques.

*Sleep hygiene (n=2, 13%)*

Sleep hygiene included interventions to improve sleep routines such as reducing caffeine and nicotine intake during evenings, waking up and going to sleep at the same time every day, and listening to calming music while lying in bed (Edinger, Wohlgemuth, Radtke, Marsh, & Quillian, 2001).

*Anger management (n=1, 7%)*

Anger management was conducted using stop-techniques and rehearsal of potentially difficult situations beforehand (Beck & Fernandez, 1998).

*Perfectionism exposure (n=1, 7%)*

Perfectionism exposure included participating in activities without conducting safety behaviors such as excessive planning (Egan, Wade, Shafran, & Antony, 2014).

**References**

Baer, R. A. (2006). Mindfulness training as a clinical intervention: a conceptual and empirical review. *Clinical Psychology: Science and Practice*, *10*(2), 125–143. https://doi.org/10.1093/clipsy.bpg015

Baikie, K. A., & Wilhelm, K. (2005). Emotional and physical health benefits of expressive writing. *Advances in Psychiatric Treatment*, *11*(5).

Barlow, D. H. (Ed.). (2008). *Clinical handbook of psychological disorders* (4th ed.). New York, NY: Guilford Publications. Retrieved from http://www.adlibris.com/se/bok/clinical-handbook-of-psychological-disorders-9781593855727

Beck, R., & Fernandez, E. (1998). Cognitive-behavioral therapy in the treatment of anger: a meta-analysis. *Cognitive Therapy and Research*, *22*(1), 63–74. https://doi.org/10.1023/A:1018763902991

Borkovec, T. D., & Costello, E. (1993). Efficacy of applied relaxation and cognitive-behavioral therapy in the treatment of generalized anxiety disorder. *Journal of Consulting and Clinical Psychology*, *61*(4), 611–619. https://doi.org/10.1037/0022-006X.61.4.611

Cernvall, M., Carlbring, P., Ljungman, G., & von Essen, L. (2013). Guided self-help as intervention for traumatic stress in parents of children with cancer: conceptualization, intervention strategies, and a case study. *Journal of Psychosocial Oncology*, *31*(1), 13–29. https://doi.org/10.1080/07347332.2012.741095

Chambers, R., Gullone, E., & Allen, N. B. (2009). Mindful emotion regulation: An integrative review. *Clinical Psychology Review*, *29*(6), 560–572. https://doi.org/10.1016/j.cpr.2009.06.005

Craske, M. G., Treanor, M., Conway, C. C., Zbozinek, T., & Vervliet, B. (2014). Maximizing exposure therapy: An inhibitory learning approach. *Behaviour Research and Therapy*, *58*, 10–23. https://doi.org/10.1016/j.brat.2014.04.006

Edinger, J. D., Wohlgemuth, W. K., Radtke, R. A., Marsh, G. R., & Quillian, R. E. (2001). Cognitive behavioral therapy for treatment of chronic primary insomnia. *JAMA*, *285*(14), 1856. https://doi.org/10.1001/jama.285.14.1856

Egan, S., Wade, T., Shafran, R., & Antony, M. (2014). *Cognitive-behavioral treatment of perfectionism.* London: Guilford Publications.

Foa, E. B., Hembree, E. A., & Rothbaum, B. O. (2007). *Prolonged exposure therapy for PTSD*. New York: Oxford University Press.

Foa, E. B., Keane, T. M., Friedman, M. J., & Cohen, J. (n.d.). *Effective treatments for PTSD*. New York: Oxford University Press.

Forster, M. (2009). *Fem gånger mer kärlek: forskning och praktiska råd för ett fungerande familjeliv*. Stockholm: Natur & Kultur.

Hawkins, A. J., Blanchard, V. L., Baldwin, S. A., & Fawcett, E. B. (2008). Does marriage and relationship education work? A meta-analytic study. *Journal of Consulting and Clinical Psychologv*, *76*(5), 723–734. https://doi.org/10.1037/a0012584

Hayes, S. C., Strosahl, K. D., & Wilson, K. G. (1999). *Acceptance and commitment therapy: An experiential approach to behavior change*. New York, NY: The Guilford Press.

Hayes, S. C., Wilson, K. G., Gifford, E. V, Follette, V. M., & Strosahl, K. D. (1996). Experiential avoidance and behavioral disorders: A functional dimensional approach to diagnosis and treatment. *Journal of Consulting and Clinical Psychology*, *64*(6), 1152–1168.

Hedman, E., Andersson, G., Andersson, E., Ljótsson, B., Rück, C., Asmundson, G. J. G., & Lindefors, N. (2011). Internet-based cognitive–behavioural therapy for severe health anxiety: randomised controlled trial. *The British Journal of Psychiatry*, *198*(3).

Hedman, E., Ljótsson, B., Rück, C., Furmark, T., Carlbring, P., Lindefors, N., & Andersson, G. (2010). Internet administration of self-report measures commonly used in research on social anxiety disorder: A psychometric evaluation. *Computers in Human Behavior*, *26*(4), 736–740. https://doi.org/10.1016/j.chb.2010.01.010

Hofmann, S. G., Sawyer, A. T., Witt, A. A., & Oh, D. (2010). The effect of mindfulness-based therapy on anxiety and depression: A meta-analytic review. *Journal of Consulting and Clinical Psychology*, *78*(2), 169–83. https://doi.org/10.1037/a0018555

Martell, C., Addis, M., & Jacobson, N. (2001). *Depression in context: Strategies for guided action*. New York, NY: Norton.

Martell, C., Dimidjian, S., & Herman-Dunn, R. (2010). *Behavioral activation for depression: a clinician’s guide.* New York: The Guilford Press.

Pennebaker, J. W. (1997). Writing about emotional experiences as a therapeutic process. *Psychological Science*, *8*(3), 162–166. https://doi.org/10.1111/j.1467-9280.1997.tb00403.x

Persons, J. (2012). *The case formulation approach to cognitive-behavior therapy.* Guilford Press, NY.

Sturmey, P. (2008). *Behavioral case formulation: a functional analytic approach.* Wiley-Blackwell.

Öst, L.-G. G. (1987). Applied relaxation: description of a coping technique and review of controlled studies. *Behaviour Research and Therapy*, *25*(5), 397–409. Retrieved from http://www.sciencedirect.com/science/article/B6V5W-4602TFR-8F/2/e69af78bc94927d41af948085b3c785c
