# Supplementary material for: An open trial of individualized face-to-face cognitive behavior therapy for psychological distress in parents of children after end of treatment for childhood cancer including a cognitive behavioral conceptualization
Source: PeerJ. 2018 Apr 11;6:e4570. doi: 10.7717/peerj.4570 (PMC5899418; doi:10.7717/peerj.4570)
Supplement: Supplemental Information 3 [file peerj-06-4570-s003.pdf]

Identification of parents' suffering related to having a child previously treated for cancer and development and evaluation of psychological treatment to reduce the psychological suffering experienced by these parents

Pediatric oncology care has improved dramatically over the past 20 years and the overall survival rate for childhood cancer is now approaching 80 % (Robison et al., 2009). Advances in treatments have ensured this development and children struck by cancer experience increasing periods of disease-free survival (Bruce, 2006). Although most children diagnosed with cancer survive the disease, challenges remain for these children and their families. A growing body of literature has shed light on the long-term physical and psychological consequences of cancer and it has been shown that childhood cancer survivors and their parents are at risk for psychological problems (Bruce, 2006).

The period after the child has recovered and no longer is on treatment is a challenging time for the family (Lindahl Norberg & Steneby, 2009). At this stage a process begins where the parents and children as well as potential siblings have to adapt to the new situation without dependence on the health care system. Many families, though relieved that the cancer is successfully treated, find it hard to return to "normal life" (Lindahl Norberg, 2008). Parents have reported perceiving less support from the health care system once their child is off treatment, although experiencing a need of continued assistance (Jackson et al., 2007). Multiple sources of stress have been identified for these families such as medical complications, fear of future long-term sequelae, relationship strains among the parents and/or the child and its potential siblings, and worries of a relapse (Lindahl Norberg, 2008; Lindahl Norberg & Steneby, 2009). Parents of children on as well as off cancer treatment are at increased risk for Posttraumatic Stress Symptoms (PTSS) (Bruce, 2006). These symptoms, such as intrusive thoughts, hyperarousal, and avoidance of anxiety arousing stimuli, are most frequent when the child is on treatment, but can persist after end of treatment. Erickson and Steiner (2000) have reported that 10 % of parents of children with cancer meet the criteria for Posttraumatic Stress Disorder (PTSD) 10 years after the child's diagnosis. In a review Bruce (2006) concludes that 10-44 % of parents of children with cancer, e.g. depending on time since diagnosis, suffers from a clinical level of PTSS.

One of our group's overall research goals is to develop psychological interventions for parents who experience a psychological suffering related to the child's cancer disease. In this project we want to explore how parents whose child has finished treatment against cancer experience their present and past life-situation, what kind of suffering, if any, they experience in connection to their child's disease, and their ideas about the future (Study A). The main part of the literature on parents' suffering related to a child's cancer disease has conceptualized the psychological symptoms that these parents report as PTSD/PTSS. We, in this project, want to explore their suffering in an inductive way by means of unstructured interviews. We also want to develop and evaluat**e** psychological treatment inspired by cognitive behavioral therapy (CBT) to help these parents to handle the psychological suffering they experience (Study B). CBT is effective in treating e.g. depression, anxiety, and PTSD (SBU, 2004; SBU 2005) but has not, to our knowledge, yet been evaluated for the psychological suffering experienced by parents of children off cancer treatment.

Aims

The aim of Study A is to identify and describe parents' suffering related to having a child treated for cancer. The aim of Study B is to develop, refine, and evaluate CBT-based treatment of psychological suffering experienced by parents of children off cancer treatment.

METHODS

Design

Study A

Study A has an explorative design. Each parent is interviewed twice by unstructured questions and interviews are audio recorded, transcribed verbatim, and analyzed with content analysis.

Study B

Study B has an uncontrolled, within group design where CBT is given to parents of children off cancer treatment. Each parent receives 10-15 sessions of face-to-face individual CBT.

Sample

The same parents participate in Study A and B. Parents are eligible if they have a child who has completed treatment for cancer at the pediatric oncology center at Akademiska Barnsjukhuset, Uppsala, 3 months to 5 years earlier; are Swedish speaking; live in Uppsala, Gävle and/or Västerås or relatively close, and experience a psychological suffering that they relate to their child's cancer disease. Parents are excluded if they suffer from a psychiatric disorder in immediate need of treatment, if they are in psychotherapy or if they have suicidal ideations (clinical judgment based on self-ratings on the Montgomery-Åsberg Depression Rating Scale (MADRS-S) (Svanborg & Åsberg, 1994) and diagnostic interview M.I.N.I). If both parents of the same child fulfill the inclusion criteria they can both be included in the study but receive treatment from different therapists.

Since we do not know what psychological suffering the participants experience or the effect of CBT on this suffering, power calculations are by necessity a crude approximation. Based on effect sizes obtained in meta-analyses of CBT for anxiety and depressive disorders (Butler, Chapman, Forman & Beck, 2006) we estimate an effect size (Cohen's d) of about 0.8 in Study B. For a power of 80 percent to detect a statistically significant difference and allowing for 25 percent dropout or non-completers, at least 20 participating parents are needed in Study B. Study A has an explorative design and inclusion will continue until saturation of the investigated phenomenon i.e. parents' suffering related to their child's cancer disease is reached. An approximation is that 20-30 parents will be included. However, the exact number of participants in the present project is not possible to decide in advance.

Treatment

The CBT interventions are based on a thorough clinical behavior analysis (Dougher, 2000) conducted during the assessment phase of the treatment, which normally extends to the first

two or three sessions. The behavior analysis guides the treatment and interventions are chosen based on this analysis and the participant’s individual needs. Three therapists work with the treatments, one licensed psychologist and two PTP-psychologists. The CBT is administered once a week at Uppsala, Gävle sjukhus, Gävle, and/or Västerås sjukhus, Västerås. Each session lasts for 45 minutes and each participant is given a maximum of 15 sessions. If a participant has remaining needs for psychological treatment after these sessions he/she is referred to the regular health care.

Data collection and instruments

Study A

Data is collected via unstructured interviews. During the interviews we ask the participants to describe their suffering in terms of thoughts and concerns about the past, present, and future related to their child’s cancer disease. The interview starts with the question: “Could you please tell me about your thoughts and concerns related to your child’s cancer disease?” Follow-up questions are posed according to the respondent’s answers. The interviews are conducted by one of the psychologists who work with the treatment in Study B. Each participant is interviewed twice. The interviews take place at Uppsala Akademiska Barnsjukhus, Uppsala, Gävle sjukhus, Gävle, and/or Västerås sjukhus, Västerås, or at another location if the participant so wishes. The interviews are audio-recorded and transcribed verbatim.

Study B

Data is collected shortly before, shortly after and 3 months after end of treatment. Participants answer questions in the following questionnaires at all assessments:

<u>The PTSD Checklist - Civilian (PCL-C):</u> The PCL-C consists of 17 items measuring symptoms of PTSD as defined in the B (re-experiencing), C (avoidance), and D (hyperactivity) criteria in DSM-IV (American Psychiatric Association, 2000). In this study the items are keyed to the child’s cancer disease. The PCL-C has shown good test-retest reliability and concurrent validity (Ruggiero, Ben, Scotti, & Rabalais, 2003; Weathers, Litz, Herman, Huska, & Keane, 1993). The PCL-C has also shown good internal consistency in the present population (Pöder et al., 2008). See Attachment 5.1.

<u>The Beck Anxiety Inventory (BAI):</u> The BAI consists of 21 items measuring symptoms of anxiety. The instrument has high internal consistency ($\alpha > .90$), good test-retest reliability ($r > .60$), and moderate to high concurrent validity ($> .50$) (Steer et. al., 2008). See Attachment 5.2.

<u>Montgomery Åsberg Depression Rating Scale Self-assessment (MADRS-S):</u> MADRS-S (Svanborg & Åsberg, 1994) consists of nine items measuring the mood over the past three days. The items consider areas such as appetite, concentration, mood, and sleep. Svanborg and Åsberg (2001) found that MADRS-S has good concurrent validity with the Beck Depression Inventory (BDI) with a correlation of .87 (Beck, Ward, Mendelson, Mock, &

Erbaugh, 1961). MADRS-S has shown good internal consistency and satisfactory test-retest reliability (Fantino & Moore, 2009). See Attachment 5.3.

Acceptance and Action Questionnaire (AAQ-II): AAQ-II (Bond & Hayes, 2005) consists of 10 items measuring experiential avoidance. The instrument has good internal consistency with α between .81 and .87 and good test-retest reliability (Bond et al., 2011). The convergent validity of the AAQ-II is good since it correlates significantly positive with measures of depression, anxiety and thought suppression (Bond et al., 2011). In the present study the instrument consists of 6 extra items measuring parents' avoidance of cancer related experiences. See Attachment 5.4.

The Penn State Worry Questionnaire (PSWQ): The PSWQ consists of 16 items measuring excessive worry (Meyer et al., 1990), has shown a high internal consistency (Cronbach's alpha .91 - .95; Meyer et al., 1990) and good test-retest reliability (r = .92; Meyer et al., 1990). PSWQ also correlates highly with other questionnaires measuring anxiety and repetitive thinking in terms of rumination (r = .57 - .78; Davey, 1993; van Rijsoort et al., 1999). See Attachment 5.5.

Rumination Scale of the Response Style Questionnaire (RSQ): The rumination scale of RSQ (Nolen-Hoeksema, 1991) measures rumination as a response to symptoms of depression. The scale consists of 22 statements and has demonstrated high internal consistency (Cronbach's alpha = .89; Nolen-Hoeksema, 1991) and good test-retest reliability (r = .80; Nolen-Hoeksema, Parker & Larson, 1994). See Attachment 5.6.

Satisfaction with Life Scale (SWLS): The SWLS measures perceived quality of life and consists of five items where the individual is asked to compare the current situation with a hypothetical standard (e.g. "I am satisfied with my life"). Individuals rate the statements on a seven-point scale from 1 = strongly disagree to 7 = strongly agree (Diener, Emmons, Larsen & Griffen 1985). The instrument has good test-retest reliability (.82) and high internal consistency (Cronbach's alpha = .87). See Attachment 5.7.

At the first assessment participants answer questions about their own demographic data and their child´s cancer disease. See Attachment 5.8 and 5.9.

A psychologist administers the Mini-International Neuropsychiatric Interview (M.I.N.I) structured diagnostic psychiatric interview for DSM-IV and ICD-10 (Sheehan, Lecrubier, Sheehan, Amorim, Javis & Weiller, 1998) to assess psychiatric disorders. At the first assessment a psychologist working with the project administers the diagnostic interview and data collection, at the following assessments another psychologist in our research group administers the diagnostic interview and the data collection. The time of each data collection is estimated to approximately 30-60 minutes, including the diagnostic interview.

Procedure

Inclusion

Brief information about the study is provided to parents of children who have finished a successful cancer treatment 3 months to 5 years previously at Akademiska Barnsjukhuset, Uppsala, in one of the following ways: (i) medical staff informs eligible parents when they come to the clinic for a checkup or (ii) medical staff contacts eligible parents that do not have a checkup scheduled at the clinic in the near future by phone and informs about the study, parents are called in alphabetical order. The medical staff briefly informs about the study and asks a potential participant if he/she is interested in getting more information, see Attachment 4.1. If a parent orally consents to having more information one of the psychologists working with the project calls the parent and provides more information about the project. Parents who experience a suffering related to the child’s disease, are not in psychotherapy and wish more information are sent written information via e-mail/mail, see Attachment 4.2, and booked for a face-to-face meeting. At the face-to-face meeting parents are once again provided written information, see Attachment 4.3, and asked about informed consent. After consent is provided parents are asked to complete the self-assessment forms and the M.I.N.I interview is administered. If uncertainty about suicidal ideations remains after this face-to-face meeting an extra phone call can be made by a licensed psychologist working in the project to make a final judgment. After this meeting (and possible the extra phone call) participation in Study A and B is offered to parents who fulfill the inclusion criteria and are not excluded (See Flow-chart, Attachment 12).

Data analyses

Study A

The audio-recorded interviews are transcribed verbatim. A computer program is used to support and facilitate the inductive thematic analysis of the interview material. Recording units are structured in categories, and the analysis continues until no new categories emerge.

Study B

Outcomes of the CBT are evaluated by statistical significance testing, estimates of effect size, reliable change indices, and the proportion of individuals achieving clinically significant change.

ETHICAL AND METHODOLOGICAL CONSIDERATIONS AND IMPORTANCE

Participation is voluntary and it is possible to withdraw from the study without any consequences at any time. The participants’ confidentiality is guaranteed and consideration is taken to participants’ integrity, dignity, and vulnerability. All data are handled according to *Personuppgiftslagen* (PUL 1998:204) and *Patientdatalagen* (208:355). The informed consent ensures that participants are well aware of the conditions of participation.

Using an uncontrolled design may be an ethical concern since parents’ efforts will yield data of less scientific value than a controlled design. However, given the paucity of knowledge about the psychological suffering of this group and the fact that we know of no other study of tailored CBT for this group, we believe that it is justified to first perform a pilot study to estimate the effectiveness of the intervention.

To date there is no evidence-based psychological treatment to offer parents of children previously treated for cancer. An overarching aim with the present study is to gain knowledge that can form the basis for a treatment program that can be evaluated in a controlled study. To develop such a treatment the long-term psychological consequences of being a parent of a child struck by cancer needs to be more fully identified, which is the aim of Study A. Study B is the first step in the process of developing a treatment for parents of children previously treated for cancer. Both studies will therefore make important contributions in the process of generating a psychological treatment that can be offered to parents suffering from the distressing experience of having a child that has gone through cancer treatment.

REFERENCES

American Psychiatric Association. (2000). *Diagnostic and statistical manual of mental disorders* (4th ed.) Washington, DC: American Psychiatric Association.

Beck, A. T., Ward, C. H., Mendelson, M., Mock, J., & Erbaugh, J. (1961). An inventory for measuring depression. *Archives of General Psychiatry, 4*(6), 561–571.

Bond, F. W., & Hayes, S. C. (2005). *Here's the AAQ-2!* Paper presented at the ACT Summer Institute, Philadelphia, PA.

Bond, F. W., Hayes, S. C., Baer, R., Carpenter, K. M., Guenole, N., Orcutt, H. K., Waltz, T., & Zettle, R. D. (2011). Preliminary psychometric properties of the Acceptance and Action Questionnaire-II: A revised measure of psychological inflexibility and experiential avoidance. *Behavior Therapy, 42*(4), 676–688.

Bruce, M. (2006). A systematic and conceptual review of posttraumatic stress in childhood cancer survivors and their parents. *Clinical Psychology Review, 26*(3), 233–256.

Butler, A.C., Chapman, J.E., Forman, E.M., & Beck, A.T. (2006). The empirical status of cognitive-behavioral therapy: A review of meta-analyses. *Clinical Psychology Review, 26*(1), 17-31.

Davey, G. C. (1993). A comparison of three worry questionnaires. *Behaviour Research and Therapy, 31*(1), 51–56.

Diener, E., Emmons, R. A., Larsen, R. J., & Griffin, S. (1985). The Satisfaction with Life Scale. *Journal of Personality Assessment, 49*(1), 71–75.

Dougher, M. J. (2000). *Clinical Behavior Analysis*. Reno: Context press.

Erickson, S. J., & Steiner, H. (2000). Trauma spectrum adaptation: Somatic symptoms in long-term pediatric cancer survivors. *Psychosomatics: Journal of Consultation Liaison Psychiatry, 41*(4), 339–346.

Fantino, B., & Moore, N. (2009). The self-reported Montgomery-Åsberg Depression Rating Scale is a useful evaluative tool in Major Depressive Disorder. *BMC Psychiatry, 9*(26). Retrieved from http://www.biomedcentral.com/bmcpsychiatry

Jackson, A.C., Stewart, H., O'Toole, M., Tokatlian, N., Enderby, K., Miller, J., & Ashley D. (2007). Pediatric brain tumor patients: Their parents' perceptions of the hospital experience. *Journal of Pediatric Oncology Nursing, 24*(2), 95-105.

Lindahl Norberg, A. (2008). Parental coping with childhood cancer. I L.K. Jacobs (red.), *Coping with Cancer.* Hauppauge, NY: Nova Science Publishers, Inc.

Lindahl Norberg, A., & Steneby, S. (2009). Experiences of parents of children surviving brain tumor: A happy ending and a rough beginning. *European Journal of Cancer Care, 18*(4), 371–380.

Meyer, T. J., Miller, M. L., Metzger, R. L., & Borkovec, T. D. (1990). Development and validation of the Penn State Worry Questionnaire. *Behaviour Research and Therapy, 28*(6), 487–495.

Nolen-Hoeksema, S. (1991). Responses to depression and their effects on the duration of depressive episodes. *Journal of Abnormal Psychology*, *100*(4), 569–582.

Nolen-Hoeksema, S., Parker, L. E. & Larson, J. (1994). Ruminative coping with depressed mood following loss. *Journal of Personality & Social Psychology, 67*(1), 92–104.

Pöder, U. (2008). Posttraumatic stress among parents of children on cancer treatment: Support, care and distress. *Dissertation from the Faculty of Medicine, Uppsala University.*

Robison, L. L., Armstrong, G. T., Boice, J. D., Chow, E. J., Davies, S. M., Donaldson, S. S., & L. K., Zeltser (2009). The childhood cancer survivor study: A National Cancer Institute-supported resource for outcome and intervention research. *Journal of Clinical Oncology, 27*(14), 2308–2318.

Ruggiero, K. J., Ben, K. D., Scotti, J. R., & Rabalais, A. E. (2003). Psychometric properties of the PTSD Checklist–civilian version. *Journal of Traumatic Stress, 16*(5), 495–502.

Sheehan D. V., Lecrubier, Y., Sheehan, K. H., Amorim, P., Janavs, J., & Weiller, E. (1998). The Mini-International Neuropsychiatric Interview (M.I.N.I.): The development and validation of a structured diagnostic psychiatric interview for DSM-IV and ICD-10. *Journal of Clinical Psychiatry, 59*(20), 22–33.

SBU - Statens beredning för medicinsk utvärdering. (2004). *Behandling av depressionssjukdomar* (SBU Publication No. 166)

SBU - Statens beredning för medicinsk utvärdering. (2005). *Behandling av ångestsyndrom* (SBU Publication No. 171)

Steer, R. A., Clark, D. A., Kumar, G., & Beck, A. T. (2008). Common and specific dimensions of self-reported anxiety and depression in adolescent outpatients. *Journal of Psychopathology and Behavioral Assessment, 30*(3), 163–170.

Svanborg, P., & Åsberg, M. (1994). A new self-rating scale for depression based on the Comprehensive Psychopathological Rating Scale. *Acta Psychiatrica Scandinavia, 89*(1), 21–28.

Svanborg, P., & Åsberg, M. (2001). A comparison between the Beck Depression Inventory (BDI) and the self-rating version of the Montgomery Åsberg Depression Rating Scale (MADRS-S). *Journal of Affective Disorders, 64*(2), 203–216.

van Rijsoort, S., Emmelkamp, P., & Vervaeke, G. (1999). The Penn State Worry Questionnaire and the Worry Domains Questionnaire: Structure, reliability, and validity. *Clinical Psychology and Psychotherapy, 6*(4), 297–307.

Weathers, F. W., Litz, B. T., Herman, D. S., Huska, J. A., & Keane, T. M. (1993). The PTSD Checklist (PCL): Reliability, validity, and diagnostic utility. *Paper presented at the Annual Meeting of International Society for Traumatic Stress Studies,* San Antonio, TX.
